# Extracorporeal membrane oxygenation as a bridge to lung transplantation: 5-year outcomes and bridge to decision in a large, older cohort

**DOI:** 10.1186/s12931-024-02968-y

**Published:** 2024-09-28

**Authors:** Jared A. Daar, Yoshiya Toyoda, Norihisa Shigemura, Sean M. Baskin, Parag Desai, Matthew Gordon

**Affiliations:** 1 Division of Cardiovascular Surgery, Temple University Hospital, Philadelphia, PA USA; 2 Department of Anesthesiology, Temple University Hospital, Philadelphia, PA USA; 3 Department of Thoracic Medicine and Surgery, Temple University Hospital, Philadelphia, PA USA

**Keywords:** Age, Bridge to transplant, ECMO, Lung transplantation, Survival

## Abstract

**Background:**

Extracorporeal membrane oxygenation (ECMO) as a bridge to lung transplantation (BTT) has expanded considerably, though evidence-based selection criteria and long-term outcome data are lacking. The purpose of this study was to evaluate whether risk factors often used to exclude patients from ECMO BTT—specifically older age and not yet being listed for transplant—are validated by long-term outcomes.

**Methods:**

To ensure minimum 5-year follow-up, a retrospective cohort study was performed of adult patients actively listed for lung transplantation at a high-volume center and bridged on ECMO between January 2012 and December 2017. Data was collected through January 1, 2023.

**Results:**

Among 50 patients bridged on ECMO, 25 survived to transplant. Median age at listing was 58 (interquartile range [IQR], 42–65) in the transplanted group and 65 (IQR, 56.5–69) in the deceased group (*P* = 0.051). One-year, 3-year, and 5-year survival were 88% (22/25), 60% (15/25), and 44% (11/25), respectively, with eight patients still living at the time of review. Median time spent at home during the year post-transplant was 340 days (IQR, 314–355). Older age at listing was a negative predictor of survival on ECMO to transplant (odds ratio 0.92 [95% confidence interval, 0.86–0.99], *P* = 0.01). Thirteen patients were placed on ECMO prior to being listed and three were listed the same day as ECMO cannulation, with 10/16 transplanted. No significant difference in post-transplant survival was found between patients placed on ECMO prior to listing (n = 10) and those already listed (n = 15) (*P* = 0.93, log-rank). Serial post-transplant spirometry up to 5 years and surveillance transbronchial biopsy demonstrated good allograft function and low rates of cellular rejection.

**Conclusions:**

In one of the oldest cohorts of ECMO BTT patients described, favorable survival outcomes and allograft function were observed up to 5 years irrespective of whether patients were previously listed or bridged to decision. Despite inherent limitations to this retrospective, single-center study, the data presented support the feasibility of ECMO BTT in older and not previously listed advanced lung disease patients.

## Background

Extracorporeal membrane oxygenation (ECMO) is used as a bridge to lung transplantation in select patients with severe cardiopulmonary failure. Over the last two decades, ECMO support for adult lung transplant candidates has expanded considerably, from less than 0.5% in 2007 [1] to 11.2% in 2021 [2]. Improvements in ECMO technology [3], increasing center experience, and the introduction of the lung allocation score system in 2005 [4], which shifted priority from waitlist time to illness severity in the allocation of donor organs, have all augmented this practice. Over time, post-transplant survival among ECMO-bridged patients has also progressively improved [5, 6].

Several dozen retrospective studies published over the last 15 years have analyzed individual transplant centers’ experience with this complex patient population [7, 8]. Consistently, favorable post-transplant survival has been reported. When comparing short- and medium-term survival to varyingly constructed control groups of non-ECMO bridged patients, some have shown no significant survival difference [9–11], while others have shown increased mortality among ECMO-bridged patients [5, 12, 13]. To date, increased center volume and experience [12, 14, 15], awake ECMO ideally with ambulation [11, 16], shorter duration of ECMO bridge [17, 18], and first instance of transplantation [19] have all been associated with superior post-transplant survival. Still, no straightforward algorithm exists to select transplant candidates for this high-risk bridging strategy. Instead, each transplant center has developed its own practice and de facto exclusion criteria when considering patients with end-stage lung disease (ESLD), in whom the only curative therapy is transplant, for ECMO.

This high-volume, single-center study represents one of the oldest cohorts of patients bridged to lung transplant on ECMO described, many of whom would be considered too high-risk by other transplant centers [20]. Included were patients not yet listed for transplant—or “bridged to decision”—at the time of decompensation requiring ECMO, a group whose status also remains controversial and is excluded by some centers [11, 21, 22]. While few centers have reported outcome data regarding ECMO-bridged lung transplant recipients beyond 36 months, we report post-transplant survival and measures of allograft function up to five years.

## Methods

This study was approved by the Temple University Institutional Review Board (Committee A1, approval #29759).

### Study protocol and patient selection

A single-center, retrospective cohort study was performed of adult patients actively listed for lung transplantation and bridged on ECMO between January 2012 and December 2017. This study period was selected to ensure minimum 5-year follow-up, with data collected through January 1, 2023. Patients were identified through the Temple University Hospital lung transplant database, with data collected from patient charts. Patients were included who were actively listed on the United Network for Organ Sharing (UNOS) transplant registry either prior to or during ECMO support. Baseline patient characteristics and data regarding patients’ hospital, ECMO, and post-discharge courses were collected.

### Survival and allograft function

Survival to transplant, discharge, and post-transplant 1-year, 3-year, and 5-year survival were analyzed. Post-transplant spirometric data and transbronchial biopsies to monitor for allograft rejection were reviewed. Biopsy specimens were graded by pathologists according to the International Society for Heart and Lung Transplantation (ISHLT) revised standardized nomenclature in the diagnosis of lung rejection [23].

### Statistical analysis

Data analysis was completed using JMP Pro, version 16.0 (SAS Institute Inc., Cary, NC). Categorical variables are presented as frequency (percentage), and continuous variables are presented as median (interquartile range [IQR]) unless otherwise specified. Categorical variables were compared using Fisher’s exact test and Pearson’s chi-square test of independence. To account for non-normal data distributions, continuous variables were compared using the nonparametric Wilcoxon rank-sum test. Kaplan–Meier analysis was used to estimate survival rates, with statistically significant differences between groups determined by the log-rank test. Multivariable logistic regression was performed using covariates that achieved a significance of *P* < 0.20 in univariable analysis. Odds ratios (OR) are presented with 95% confidence intervals (CI). Two-sided *P* values are reported, with a *P* value < 0.05 considered statistically significant.

## Results

### Patient characteristics

**Fig. 1:**
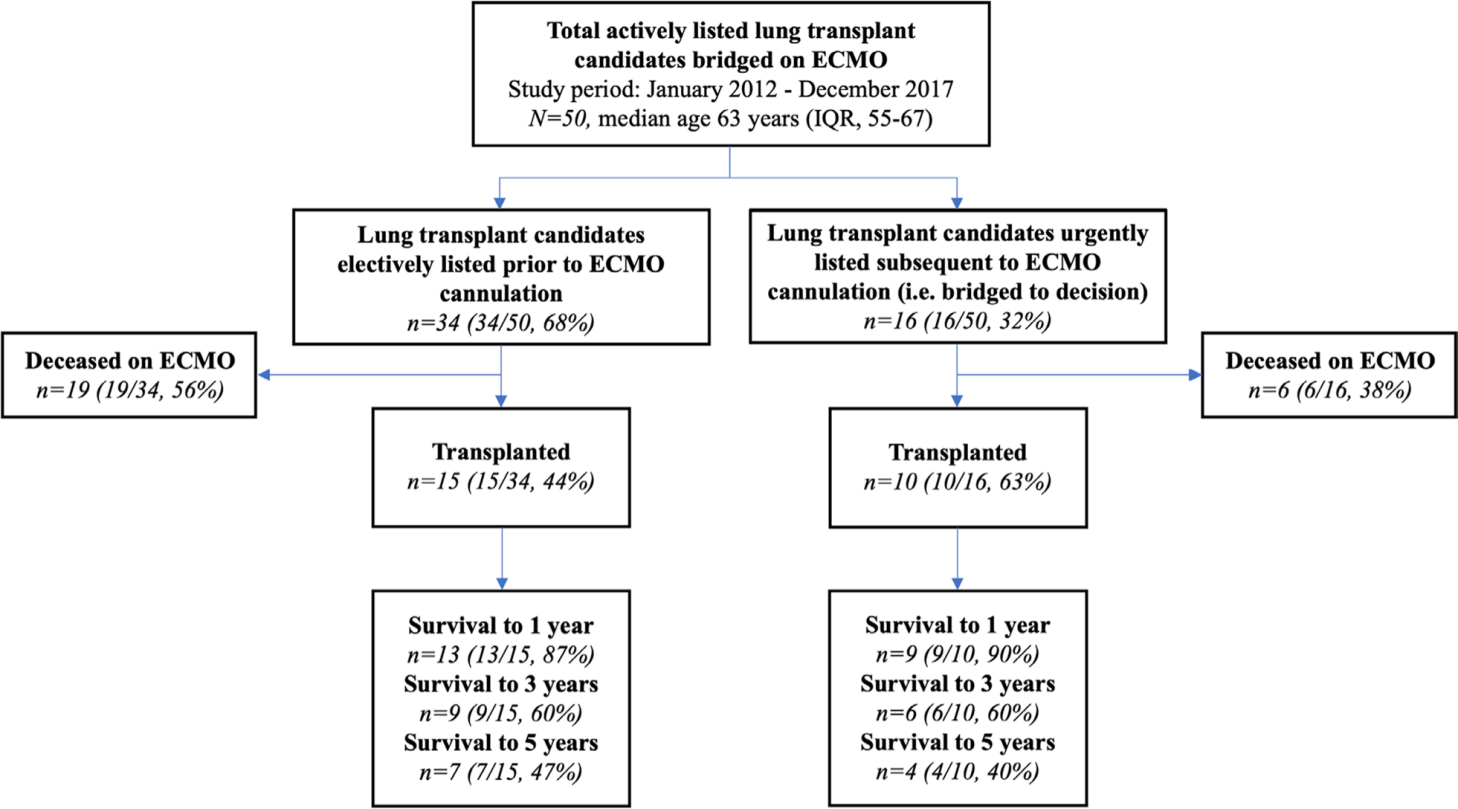
Flow diagram of the study cohort, with subdivision of patients listed electively prior to extracorporeal membrane oxygenation (ECMO) cannulation vs. urgently following ECMO cannulation (i.e. bridged to decision). Survival is indicated at various points pre- and post-transplantation. IQR: interquartile range

Fifty patients met the inclusion criteria, 27 women and 23 men (Table 1*, *Fig. 1). Of these, 25 (14 women, 11 men) survived to lung transplant. Median age at listing was 58 (IQR, 42–65) in the transplanted group and 65 (IQR, 56.5–69) in the deceased group (*P* = 0.051). Idiopathic pulmonary fibrosis was the predominant transplant diagnosis, with 19 patients in each group. No significant difference in lung allocation score (*P* = 0.25), underlying transplant diagnosis (*P* = 0.77), sex (*P* = 0.78), or blood group (*P* = 0.93) was observed between those transplanted and those that died before transplant.

**Table 1:** Patient characteristics

	Transplanted (n = 25)	Deceased pre-transplant (n = 25)	Total (N = 50)	*P* value
Age at listing, median (IQR)	58 (42–65)	65 (56.5–69)	63 (55–67)	0.051
Sex, n (%)				0.78
Female	14 (56)	13 (52)	27 (54)	
Male	11 (44)	12 (48)	23 (46)	
BMI, median kg/m^2^ (IQR)	28.4 (21.6–32.5)	28.9 (24–31.9)	28.5 (21.9–32)	0.68
Height, median cm (IQR)	165 (157–178)	165 (161–175)	165 (160–178)	0.98
Transplant diagnosis, n (%)				0.77
Idiopathic pulmonary fibrosis	19 (76)	19 (76)	38 (76)	
Primary pulmonary hypertension	2 (8)	2 (8)	4 (8)	
Sarcoidosis	–	2 (8)	2 (4)	
Re-transplant/Primary graft failure	1 (4)	1 (4)	2 (4)	
Acute interstitial pneumonia	1 (4)	–	1 (2)	
COPD	–	1 (4)	1 (2)	
Lymphocytic interstitial pneumonia	1 (4)	–	1 (2)	
Mixed connective tissue disease	1 (4)	–	1 (2)	
Blood group, n (%)				0.93
A	9 (36)	10 (40)	19 (38)	
B	4 (16)	5 (20)	9 (18)	
AB	1 (4)	1 (4)	2 (4)	
O	11 (44)	9 (36)	20 (40)	
LAS at time of transplant or death, median (IQR)	88.7 (85.2–90.4)	85.6 (82.4–90.4)	87.6 (84.4–90.4)	0.25
PRA, median (IQR)^a^	0% (0–10%)	0% (0–10%)	0% (0–10%)	0.53
PaO2/FiO2 at ECMO initiation, median (IQR)^b^	73 (58.5–97.5)	66 (52–80)	68.5 (57–84.5)	0.24
ECMO prior to listing, n (%)	9 (36%)	4 (16%)	13 (26%)	0.20
ECMO same day as listing, n (%)	1 (4%)	2 (8%)	3 (6%)	1.00
Transplant type, n (%)				
Double lung	22 (88)			
Single lung	2 (8)			
Heart + Double lung	1 (4)			

IQR: interquartile range; BMI: body mass index; COPD: chronic obstructive pulmonary disease; LAS: lung allocation score; PRA: panel-reactive antibodies; PaO2: partial pressure of oxygen in arterial blood; FiO2: fraction of inspired oxygen; ECMO: extracorporeal membrane oxygenation

^a^PRA never obtained for one patient in the deceased group (n = 24)

^b^Pre-ECMO arterial blood gas unavailable for 2 patients in the deceased group (n = 23)

### Hospital and ECMO course

Total hospital length of stay ranged from 4 to 284 days (Table 2). A significant difference was observed in total hospital length of stay between groups, median of 73 days (IQR, 45.5–122.5) in the transplanted group vs. 48 days (IQR, 22–74.5) in the deceased group (*P* = 0.02). A significant difference was also noted in time spent in Status-7 (temporarily inactivated from listing, typically due to being too ill), with a median of 0 days (IQR, 0–3) in the transplanted group and 17 days (IQR, 2–22.5) in the deceased group (*P* < 0.001). The median time spent on ECMO was 13 days (IQR, 5.5–53) among transplanted patients and 18 days (IQR, 11–48) among deceased patients (*P* = 0.50), with a range of 1 to 170 days. Venovenous ECMO was the predominant configuration used (19 and 16 cases in each group, respectively), with no significant difference in ECMO configuration between groups (*P* = 0.56). Twenty patients in each group and 80% overall were unable to be weaned from mechanical ventilation while on ECMO, typically due to refractory hypoxemia.

**Table 2:** Hospital and ECMO course

	Transplanted (n = 25)	Deceased pre-transplant (n = 25)	Total (N = 50)	*P* value
Hospital length of stay, median days (IQR)				
Total, hospital	73 (45.5–122.5)	48 (22–74.5)	57.5 (33–107)	0.02
Total, ICU	41 (24.5–90)	33 (16.5–58)	36.5 (17–75)	0.24
Post-transplant, hospital	39 (25–54.5)			
Post-transplant, ICU	17 (9–30.5)			
Days actively listed, median (IQR)	17 (4–36)	27 (12–72)	19 (10–63)	0.36
Days Status-7, median (IQR)	0 (0–3)	17 (2–22.5)	2.5 (0–21)	< 0.001
ECMO configuration, n (%)^a^				0.56
VV	19 (76)	16 (64)	35 (70)	
VA or VVA	3 (12)	6 (24)	9 (18)	
VV + RVAD	3 (12)	3 (12)	6 (12)	
ECMO Duration, median days (IQR)				
Total	13 (5.5–53)	18 (11–48)	16.5 (8–50)	0.50
Pre-transplant	10 (3–46.5)			
Post-transplant	0 (0–3)			
Mechanical ventilation while on ECMO, n (%)	20 (80)	20 (80)	40 (80)	1.00
Ventilator days, median (IQR)				
Pre-ECMO	1 (0–5)	1 (0–6.5)	1 (0–5)	0.79
Post-transplant	27 (8.5–43.5)			
Blood products on ECMO, median (IQR)^b^				
Total pRBCs	10 (3.5–38)	15.5 (10.5–27.5)	13 (6.5–31)	0.21
Average daily pRBCs	0.6 (0.4–1)	0.7 (0.4–1.3)	0.6 (0.4–1)	0.85
Total platelets	1 (0–2)	2 (0–5)	2 (0–4)	0.21
Total FFP	0 (0–2)	2 (0–5.5)	1 (0–4)	0.34
Total cryoglobulin	0 (0–2)	0 (0–0)	0 (0–1.5)	0.048
Total blood products	12 (5–43.5)	21.5 (13.5–35.5)	17 (8–37)	0.16
Average total daily blood products	0.8 (0.4–1.3)	0.9 (0.4–1.8)	0.8 (0.4–1.5)	0.67
Complications on ECMO, n (%)^c^				
Massive bleed (6 + pRBCs given)	4 (16)	10 (40)	14 (28)	0.11
Renal replacement therapy	2 (8)	6 (24)	8 (16)	0.25
Stroke	1 (4)	1 (4)	2 (4)	1.00
Thrombosis	5 (20)	4 (16)	9 (18)	1.00
Cardiac arrest	1 (4)	4 (16)	5 (10)	0.35
Thrombocytopenia	20/21 (95)	25/25 (100)	45/46 (98)	0.46
Hemolysis	21/21 (100)	25/25 (100)	46/46 (100)	1.00

ECMO: extracorporeal membrane oxygenation; IQR: interquartile range; ICU: intensive care unit; VV: venovenous; VA: venoarterial; VVA: veno-venoarterial; RVAD: right ventricular assist device; pRBC: packed red blood cells; FFP: fresh frozen plasma

^a^Whenever ECMO was escalated from a primarily respiratory to hemodynamic support configuration, the highest level of hemodynamic support was reported, such that: VV < VV + RVAD < VA or VVA

^b^Blood product information incomplete and excluded for 1 patient in the deceased group (n = 24)

^c^Thrombocytopenia and hemolysis data excluded for four patients transplanted within 1 day of ECMO cannulation (N = 46)

One patient was treated for acute antibody-mediated rejection (AMR) following transplant and was discharged. Two transplanted patients died before hospital discharge, one from complications of hyperacute AMR and one from cardiac arrest several weeks post-operatively.

### Complications and predictors of survival

Anemia while on ECMO was common, with all but 3 patients (94%) requiring packed red blood cell (pRBC) transfusion and no significant difference in median total pRBCs (*P* = 0.21) or total blood products (*P* = 0.16) transfused between groups. No significant difference in incidence of any specific ECMO complication, including stroke (*P* = 1.00), thrombosis (*P* = 1.00), massive bleed requiring 6 + pRBCs (*P* = 0.11), renal replacement therapy (*P* = 0.25), or cardiac arrest (*P* = 0.35) was observed. In a multivariable logistic regression analysis, only older age at listing (OR 0.92 [95% CI, 0.86–0.99], *P* = 0.01) and massive bleed (OR 0.41 [95% CI, 0.14–0.98], *P* = 0.04) were identified as significant negative predictors of survival to transplant (Table 3).

**Table 3:** Multivariable logistic regression to predict survival on ECMO to lung transplantation

Predictor	OR (95% CI)	*P* value
Cardiac arrest	0.39 (0.08–1.31)	0.13
Renal replacement therapy	0.49 (0.15–1.27)	0.14
Massive bleed (6 + pRBCs given)	0.41 (0.14–0.98)	0.04
Days Status-7	0.97 (0.94–1.01)	0.07
Age at listing	0.92 (0.86–0.99)	0.01

ECMO: extracorporeal membrane oxygenation; OR: odds ratio; CI: confidence interval; pRBC: packed red blood cells

### Post-hospital course and allograft function

**Fig. 2:**
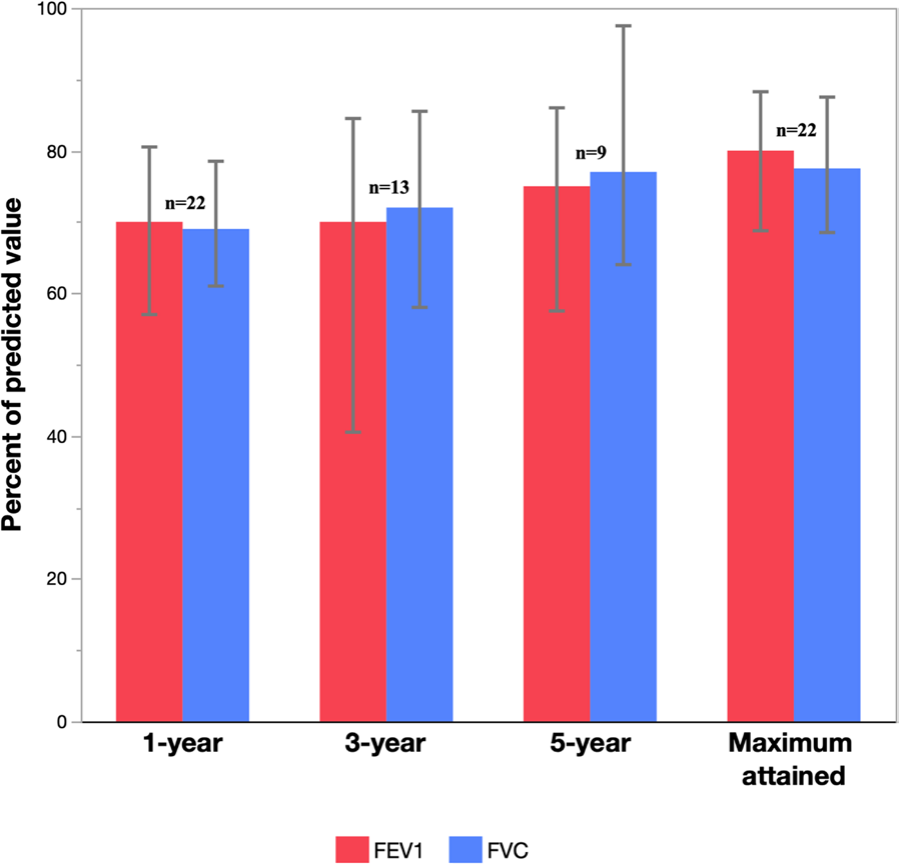
Post-transplant spirometric data, collected at approximate 1-year (n = 22), 3-year (n = 13), and 5-year (n = 9) intervals. Spirometry was never completed for three transplanted patients. The maximum attained forced expiratory volume in one second (FEV1) and forced vital capacity (FVC) at any time post-transplant are presented as proxies for each patient’s post-transplant baseline allograft function (n = 22). Median values are shown with error bars representing interquartile ranges

The median time spent at home during the year post-transplant was 340 days (IQR, 314–355), with a median hospital readmission time of 6 days (IQR, 0–16) (Table 4). In post-transplant spirometry conducted at regular intervals (n = 22), median maximum attained forced expiratory volume in one second (FEV1) and forced vital capacity (FVC) were 80% (IQR, 69–87%) and 77.5% (IQR, 69–87%) of the predicted values, respectively. Median (IQR) FEV1 and FVC at 1-year (n = 22), 3-year (n = 13), and 5-year (n = 9) intervals are displayed in Fig. 2.

**Table 4:** Post-transplant survival and time home

Survival post-transplant	
1-year survival, n (%)	22 (88%)
3-year survival, n (%)	15 (60%)
5-year survival, n (%)	11 (44%)
Post-transplant discharge year 1, median (IQR)^a^	
Rehab days	16 (8–21)
Hospital readmission days	6 (0–16)
Days home	340 (314–355)

IQR: interquartile range

^a^Among patients transplanted, two died prior to hospital discharge (n = 23)

Surveillance transbronchial biopsy with ISHLT grading was performed at least once in 21/25 transplanted patients, with a total of 50 biopsies reviewed (Table 5). Biopsies were performed with varying frequency, but 82% occurred within 24 months of transplant. One specimen was graded A1 and another A1-A2, suggesting minimal and minimal-to-mild evidence of acute cellular rejection, respectively. Three specimens were graded B1R, indicating low grade airway inflammation, and one was graded C1, indicating the presence of chronic airway rejection.

**Table 5:** Surveillance transbronchial biopsies and allograft rejection^a^

Total transbronchial biopsies (TBBx) performed, n	50
Timing of TBBx	
≤ 12 months after transplant, n (%)	28 (56)
13–24 months after transplant, n (%)	13 (26)
> 24 months after transplant, n (%)	9 (18)
Number of TBBx performed per patient	
Zero, n (%)	4 (16)
One, n (%)	6 (24)
Two, n (%)	6 (24)
Three or more, n (%)	9 (36)
ISHLT grades of TBBx specimens	
A1 or A1-A2, n (%)	2 (4)
B1R, n (%)	3 (6)
C1, n (%)	1 (2)
Ungradeable—Ax/Bx/Cx, n (%)	5 (10)/10 (20)/7 (14)
Patients treated for acute or hyperacute antibody-mediated rejection, n (%)	2 (8)

IQR: interquartile range; ISHLT: International Society for Heart and Lung Transplantation

^a^Transbronchial biopsy was never performed for four transplanted patients (n = 21)

### Survival and bridge to decision

Among 25 patients transplanted, 1-year, 3-year, and 5-year survival were 88% (22/25), 60% (15/25), and 44% (11/25), respectively (Table 4). Eight patients were still living at the time of review.

**Fig. 3:**
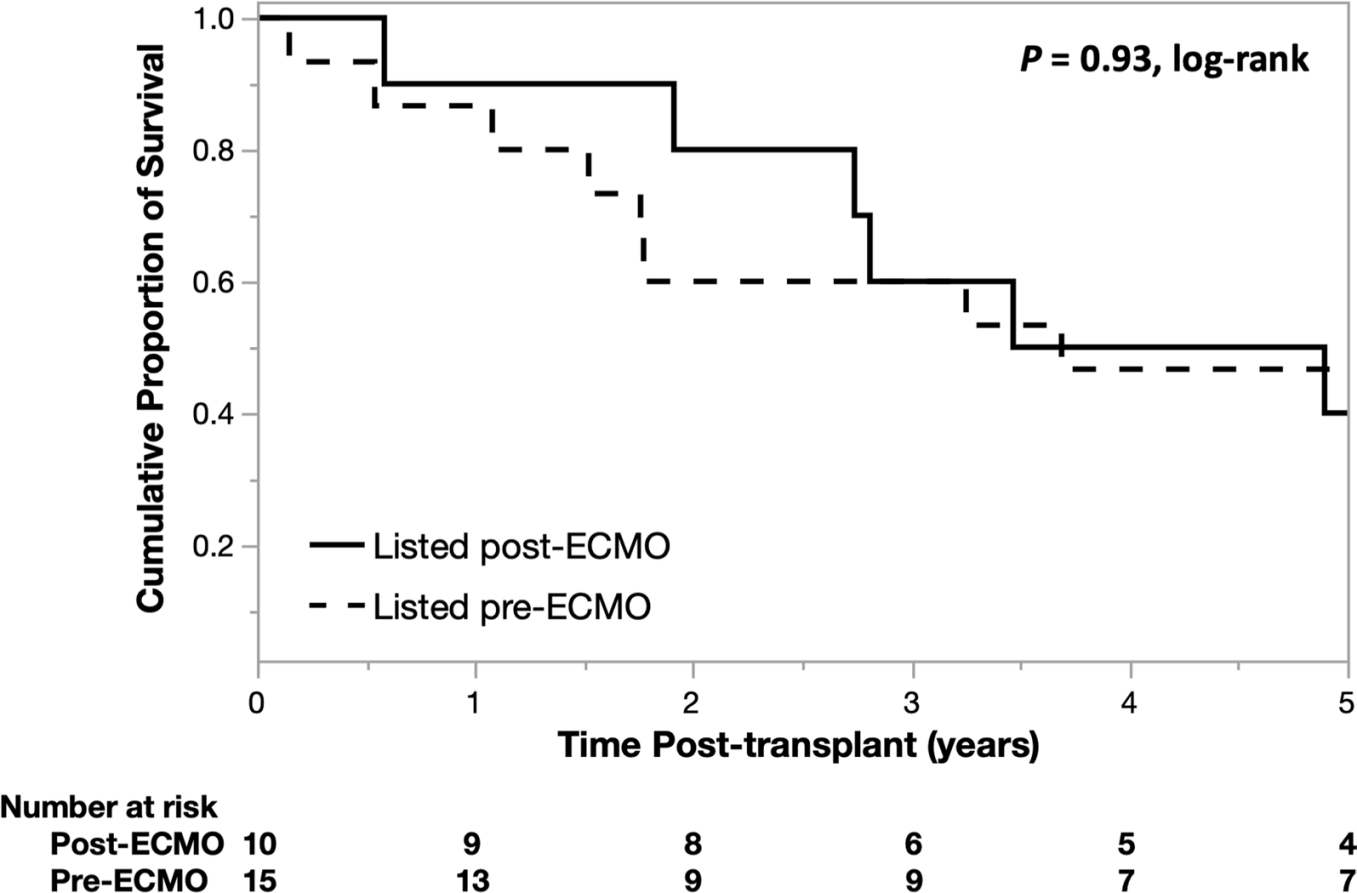
Kaplan–Meier survival estimates of transplanted patients placed on extracorporeal membrane oxygenation (ECMO) prior to active listing (n = 10), i.e. bridged to decision, compared to patients placed on ECMO who were already listed (n = 15)

Thirteen patients were placed on ECMO prior to being actively listed on UNOS, and 9/13 survived to transplant, with four still living at the time of review. Three patients were listed on UNOS the same day as ECMO cannulation (two having previously completed evaluation facilitating urgent listing, one completing the remaining studies the same day following cannulation), with one surviving to transplant and deceased at the time of review. When comparing transplanted patients placed on ECMO after listing (n = 15) with those placed on ECMO either prior to listing or the same day (n = 10), no significant difference in post-transplant survival was observed (*P* = 0.93, log-rank, Fig. 3).

## Discussion

From January 2012 to December 2017, 50 lung transplant candidates with a median age at listing of 63 years (IQR, 55–67) were bridged on ECMO at our center (including 16 patients not listed at the time of cannulation), and 25 survived to transplant. Within this cohort, 1-year survival (88%) closely approximated the most recently published national rate (87.8%) for all adult lung transplant recipients [24]. Most important, these patients spent the vast majority of time at home (median 340 days [IQR, 314–355]) in the year following transplant. Favorable 3-year (60%) and 5-year (44%) survival were also observed, though these rates fell below those of similarly aged recipients nationwide (approximately 70% and 57%, respectively, for recipients 60 and older) [24] as well as the few other ECMO-bridged cohorts reporting 5-year survival (noting that none included patients near our cohort’s extreme of age, as detailed below). Altogether, favorable survival outcomes and allograft function were observed up to 5 years irrespective of whether patients were previously listed or bridged to decision. Despite inherent limitations, this study—encompassing one of the oldest cohorts of ECMO-bridged lung transplant candidates described—supports the feasibility of ECMO as a bridge to lung transplantation in older and not previously listed advanced lung disease patients.

Prior to this study, limited long-term survival data beyond 36 months have been reported in ECMO-bridged lung transplant recipients. When compared to cohorts from four other high-volume transplant centers we are aware of that have reported contemporaneous 5-year survival in this unique patient population, first, our patients were substantially older, with a median age at listing of 63 years (IQR, 55–67). By contrast, the Medical University of Vienna reported 60% 5-year survival among 70 patients bridged from 2010–2017 with a median age of 36 (IQR, 8–68); the University of Pittsburgh 66% 5-year survival among 49 patients from 2008–2015 with a mean age of 44.8 (SD ± 13.5); Asan Medical Center 62% 5-year survival among 78 patients from 2008–2018 with a median age of 55 (IQR, 41–62); and the University of Toronto 48% 5-year survival among 71 patients from 2006–2016 with a median age of 38 (range, 18–62) [5, 10, 18, 19]. Second, our patients spent longer on ECMO pre-operatively (median 13 days [IQR, 5.5–53] compared to 5 [IQR, 3–8] in the Vienna cohort, 13 [IQR, 7–19] in the Asan cohort, and 10 [range, 0–95] in the Toronto cohort; data unavailable for the Pittsburgh cohort), a demonstrated risk factor for lower post-transplant survival [17, 18]. Third, our patients required more mechanical ventilation and sedation on ECMO due to refractory hypoxemia and inability to liberate from the ventilator, additional factors known to worsen deconditioning and correlate with lower post-transplant survival [16]. In sum, while our patients’ 5-year survival was lower than elsewhere, we consider this outcome favorable given an older, higher risk group of patients who required pre-transplant ECMO and mechanical ventilation for longer.

A substantial number of patients (n = 16) at our center were placed on ECMO either prior to active listing on UNOS (13/16) or the same day as listing (3/16). This represents a departure from some high-volume centers, whose protocols require a patient to be listed prior to offering ECMO support [11]. Controversy exists surrounding this practice of “bridging to decision” with some suggesting it should only be considered at experienced, high-volume lung transplant centers capable of performing expedited transplant evaluations [21, 22]. A key element of this argument is the demonstrated survival advantage for ECMO-bridged patients treated at high-volume versus low- and medium-volume centers [12, 14, 15]. A bias may also exist on some selection committees, however, toward deferring listing for patients becoming progressively sicker.

Our data show success both in completing expedited evaluations and bridging these patients to transplant (10/16, 63%). One-year, 3-year, and 5-year post-transplant survival were 90% (9/10), 60% (6/10), and 40% (4/10), respectively, in this subgroup (Fig. 1)*.* These results corroborate and extend those of Kukreja and colleagues, who have also shown success bridging emergently waitlisted patients (12/20, 60%) to transplant [25]. Some degree of selection bias is inevitable in our data, since not all ESLD patients who may have been placed on ECMO and considered for transplant were systematically identified and reviewed. Whereas Kukreja et al*.* reported 14/34 patients who failed to be listed following ECMO cannulation, we only analyzed patients who were eventually listed. Nonetheless, the lack of a significant post-transplant survival difference between these patients and those bridged post-listing (*P* = 0.93, log-rank, Fig. 3) offers further support to this practice.

The mortality rate on ECMO prior to transplant (50%) at our center was also higher than elsewhere. Taken as a proxy for physiologic reserve, the older age of our patients—which was found to be a significant negative predictor of survival to transplant (OR 0.92 [95% CI, 0.86–0.99], *P* = 0.01)—likely accounts somewhat for the higher pre-transplant mortality observed. A systematic review of 10 single-center studies published between 2010 and 2013 found pre-transplant mortality on ECMO to range from 17 to 50% [7]. In more recent studies, pre-transplant mortality has been (in descending order of cohort size) 42% (51/121) [11], 47% (37/78) [18], 11% (8/71) [19], 10% (7/70) [5], 32% (20/62) [25], 13% (4/30) [26], 55% (11/20) [27], and 0% (0/12) [28]. Altogether, these results reflect a wide range of center experiences, with no clear indication that high volume alone guarantees successful bridge to transplant.

We used a combination of surveillance transbronchial biopsy and post-transplant spirometry to monitor for rejection and detect changes in lung allograft function over time. Institutional practice regarding transbronchial biopsy fluctuated within the study period, with 4 transplanted patients never undergoing biopsy and the remainder with varying frequency. Of the 50 biopsies performed, however, low rates of acute cellular rejection were observed. One patient was treated for AMR and discharged, and just two other patients received “A1” and “A1-A2” grades suggesting minimal to mild evidence of acute cellular rejection. One patient, who experienced hyperacute AMR and died post-operatively despite treatment, was known to be extremely high risk (panel-reactive antibodies of 92%) and was transplanted following an extensive risk–benefit discussion with the patient and family.

The ISHLT defines a patient’s post-transplant baseline allograft function as the average of the highest two FEV1 values taken at least 3 weeks apart. In turn, a sustained decline ≥ 20% in FEV1 ± FVC from baseline may signal the development of chronic lung allograft dysfunction [29]. Likewise, a concurrent decline in FEV1 and FVC may portend higher 1-year and 5-year mortality [30]. We report the maximum FEV1 and FVC attained by each patient (80% [IQR, 69–87%] and 77.5% [IQR, 69–87%] of the predicted values, respectively) as a proxy for their post-transplant baseline, with serial measurements at 3 and 5 years among surviving patients (Fig. 2). The ISHLT method yields a comparable median FEV1 of 78.5% (IQR, 67–85.5%) predicted. Most important, these results demonstrate strong baseline allograft function across the cohort of transplanted patients who completed spirometry (22/25). While a decline ≥ 20% in FEV1 from the calculated baseline occurred in 10/22 patients (45%), with a concurrent decline in FVC in 9/22 patients (41%), this appears consistent with lung transplant recipients globally, of whom ~ 41% develop bronchiolitis obliterans syndrome by 5 years [31].

Despite an abundance of data regarding ECMO bridge to transplant, one of the major unresolved issues within the field remains how best to select patients most likely to benefit from this strategy. Tipograf et al*.*, reporting on one of the largest single-center cohorts to date (N = 121), found only ambulation while on ECMO to be significant in predicting a successful bridge to transplant [11]. Other factors including renal replacement therapy predicted death before transplant, while age was not a significant predictor. By contrast, our data did not find renal replacement therapy to be a significant predictor of survival to transplant (noting this may partly be attributable to a lack of statistical power due to sample size), while older age at listing was a significant negative predictor. While data from more centers are needed to validate our finding, this may reflect a difference observed at the upper extreme of age: whereas Tipograf’s cohort had a median age of 44 (IQR, 30–58), ours was substantially higher at 63 (IQR, 55–67).

Our findings are also consistent with a recent UNOS database analysis that found inferior post-transplant survival in 159 ECMO-bridged lung transplant recipients aged 65 and older from 2008–2022 when compared to non-ECMO-bridged recipients [32]. Despite inferior survival, however, over half of the transplanted patients in this cohort as in ours were still living at 3 years post-transplant. Rather than simplifying the matter, this merely raises the question: What degree of impaired survival should deter the use of ECMO in this age group?

Another significant difference between those transplanted and those who died was time spent in Status-7, or temporarily inactive on UNOS: 0 days (IQR, 0–3) in the transplanted group vs. 17 days (IQR, 2–22.5) in the deceased group (*P* < 0.001). While this finding makes intuitive sense—patients too sick to remain actively listed are less likely to be transplanted—it may also inform transplant committees weighing difficult decisions about continuing a patient’s prolonged Status-7 on ECMO versus permanent delisting and palliative care.

Limitations of this study include a small single-center population, which limits the generalizability of results. Our cohort, however, is relatively large compared to most within the literature, with inherent limitations to ECMO and transplantation research often necessitating cumulative data from individual centers. The study also lacks a comprehensive control group for extensive comparison with non-ECMO-bridged lung transplant recipients. The purpose of this study, however, was not to compare outcomes among ECMO-bridged and non-ECMO-bridged lung transplant recipients (an analysis that has been conducted many times previously). Our aim was rather to describe a novel cohort of substantially older and, in many cases, not previously listed ECMO-bridged lung transplant candidates to evaluate the feasibility of ECMO bridge to transplant in these understudied, occasionally excluded subgroups. Additional patients with advanced lung disease who may have been placed on ECMO and considered for lung transplantation but never listed were not systematically identified or reviewed, limiting claims regarding the overall success of bridging to decision. The few transbronchial biopsies taken beyond 24 months also limit the evaluation of longer-term cellular rejection. Some degree of era bias in ECMO and lung transplantation practices is introduced by examining a study population from 2012–2017. This limitation was unavoidable, however, to ensure a minimum of 5-year outcome data (and if anything, outcomes in these subgroups are likely to have improved since 2017 with increasing transplant center experience and refinements in practice). Last, the analysis was focused entirely on lung transplant patient characteristics and hospital course, without consideration of intraoperative techniques or organ donor characteristics.

The broader implications of offering ECMO as a bridge without certain success, particularly among older patients who may have lower odds of survival to transplant, deserve continued attention [33, 34]. In our own intensive care units, cases of prolonged ECMO where patients became too sick for transplant have been among the most complex and difficult for patients, families, and providers alike. Ultimately, we feel the favorable outcomes and post-transplant quality of life evidenced by this study support the use of ECMO in select, older ESLD patients with a reasonable chance of transplant and long-term survival.

## Conclusions

In one of the oldest cohorts of patients undergoing ECMO as a bridge to lung transplantation described, 1-year survival closely approximated that of all adult lung transplant recipients nationwide, with quality of life evidenced by time spent at home in the year following transplant. Three-year and 5-year survival were also favorable in this cohort, though lower than national rates among similarly aged recipients and other contemporaneous ECMO-bridged cohorts (noting that these few comparator studies tended to include much younger patients). Post-transplant spirometry demonstrated good allograft function with low rates of cellular rejection on transbronchial biopsy. Older age at listing was a negative predictor of survival to transplant on ECMO, with relatively high pre-transplant mortality observed. Among older patients surviving to transplant, however, favorable long-term survival was observed irrespective of whether patients were actively listed prior to ECMO or bridged to decision followed by listing and transplant. Despite inherent limitations to this and prior single-center studies, the data presented support the feasibility of ECMO as a bridge to lung transplantation in older and not previously listed advanced lung disease patients.

## Data Availability

The data that support the findings of this study are not publicly available due to privacy or ethical restrictions, but may be made available on reasonable request from the authors.

## Abbreviations

AMR: Antibody-mediated rejection
CI: Confidence interval
ECMO: Extracorporeal membrane oxygenation
ESLD: End-stage lung disease
FEV1: Forced expiratory volume in one second
FVC: Forced vital capacity
IQR: Interquartile range
ISHLT: International Society for Heart and Lung Transplantation
OR: Odds ratio
pRBC: Packed red blood cells
UNOS: United Network for Organ Sharing
